# Morphometric characterization of Holocene mandibles expands the ecological baseline for understanding gibbon extinction dynamics

**DOI:** 10.1098/rsos.242065

**Published:** 2025-06-25

**Authors:** Samuel T. Turvey, Alejandra Ortiz, Matthew Granger, Selina Brace, Rasmus Amund Henriksen, Qingping Yang, Tuấn Anh Nguyễn, Laura T. Buck, Heidi Ma, James P. Hansford, Thomas Booth, Helen J. Chatterjee, Pengfei Fan, Xi Chen

**Affiliations:** ^1^ Institute of Zoology, Zoological Society of London, London, UK; ^2^ Department of Anthropology, New York University, New York, NY, USA; ^3^ Arizona State University, Tempe, AZ, USA; ^4^ Department of Science, The Natural History Museum, London, UK; ^5^ University of Copenhagen, Kobenhavn, Denmark; ^6^ Guangxi Institute of Cultural Relic Protection and Archaeology, Nanning, People’s Republic of China; ^7^ Faculty of Environmental Sciences, University of Science, Vietnam National University, Hanoi, Vietnam; ^8^ Liverpool John Moores University, Liverpool, UK; ^9^ University College London, London, UK; ^10^ School of Life Sciences, Sun Yat-Sen University, Guangzhou, People’s Republic of China; ^11^ Department of Cultural Heritage and Museology, Nanjing Normal University, Nanjing, People’s Republic of China

**Keywords:** cao vit gibbon, conservation palaeobiology, geometric morphometrics, historical baselines, *Nomascus nasutus*, refugee species

## Abstract

Human activities have driven biodiversity loss for millennia, and conservation of ‘refugee species’ that survive as remnant populations requires insights from historical baselines. However, reconstructing the past distribution and ecology of such species is challenging due to data limitations with specimen-based archives. Here, we assess the taxonomic identity of two gibbon mandibles from the Wumingshan Neolithic site in Guangxi, China. Although ancient DNA extraction was unsuccessful, a suite of linear and geometric morphometric analyses using dental and mandibular characters reveals that these mandibles fall within or close to variation shown by extant Chinese *Nomascus* gibbons and can be assigned to the cao vit gibbon *N. nasutus*. This is now one of the world’s rarest mammals, with a surviving population of 74 individuals in one site. Comparative assessment of bioclimatic, abiotic and anthropogenic parameters for Wumingshan and other sites where *N. nasutus* historically occurred reveals the species was formerly a landscape generalist but is now restricted to a high-elevation refugium with reduced human pressures. Our multidisciplinary analyses provide a new baseline on niche requirements and vulnerability for *N. nasutus* with implications for population management, demonstrating the importance of integrating environmental archives into conservation planning.

## 1. Introduction

Conservation of threatened species requires an evidence-based approach, where management decisions are informed by empirical data on key parameters such as species’ ecological requirements [1]. Most conservation evidence utilizes recent baselines derived from ecological data-collection approaches [2,3]. However, human activities have driven biodiversity loss for millennia, meaning that conservation inferences based only upon modern states may be incomplete and biased [4]. For example, many species have experienced historical human-caused range collapse, and survive as ‘refugee species’ in sites that do not exhibit the full range of environmental conditions they can tolerate [5,6]. Such sites can represent ecologically suboptimal or marginal conditions where survival reflects spatial heterogeneity in threats rather than habitat quality, such as high-elevation or otherwise inaccessible refugia where human access is restricted by landscape conditions [7–9]. Using parameters associated with survival of remnant populations to guide conservation can therefore lead to erroneous assumptions about their ecological requirements and tolerance to change, and can promote conservative or inappropriate management targets. While it may not be possible to restore species to regions from which they have been extirpated (e.g. due to human-driven transformation of natural habitats), reconstructing the environmental determinants of threatened species’ past distributions, and their resilience or vulnerability across different landscapes over time, can provide new predictive insights for conservation planning [10,11].

However, reconstructing the past distribution and ecology of refugee species can prove challenging. Environmental archives are available for many systems and can potentially provide insights into past biodiversity states, but these archives vary in both quantity and quality, such as in taxonomic and geographic representation and resolution [12]. Specimen-based archives are usually morphologically incomplete, and zooarchaeological and fossil samples, which often pre-date major human impacts to priority systems, typically only constitute preserved hard tissues of varying diagnostic status [13]. For species reduced to tiny surviving populations, comparative modern material is often also limited, compounding the problems of identifying historical specimens. Evaluating the conservation information-content of environmental archives for threatened species therefore requires critical appraisal of available data, often using multiple approaches.

Evidence-based conservation is particularly urgent for eastern and southeast Asia, as this biodiversity-rich region is experiencing extreme resource overexploitation and habitat conversion, and contains the world’s highest proportions of threatened terrestrial vertebrates [14]. Although availability of environmental archives varies across this region, rich historical, zooarchaeological and fossil archives exist in China, which has enabled reconstruction of long-term faunal dynamics and biodiversity responses to past human pressures [15,16]. These archives have also identified several Asian mammals as refugee species that persist today in reduced areas of their former ranges, which was not apparent from modern baselines alone [17–20].

Hylobatids (gibbons and siamangs) are arboreal small apes restricted to eastern and southeast Asia, and all 20 living species are now threatened [21]. Historical records indicate that gibbons formerly occurred across eastern, central and southern China, but nearly all Chinese populations are now extinct [17]. Remnant populations of four species still persist in fragmented habitats in southwest China [22]. At least two other extant species also occurred in southwest China until the twentieth century [22], and a central Chinese species (*Junzi imperialis*) is now globally extinct [23]. Pleistocene fossils represent additional extinct Chinese species, including *Bunopithecus sericus* [24] and other taxa known only from teeth [25]. However, the taxonomic identities and distributions of most extinct Chinese gibbons are unclear, as primates are underrepresented in Asian Quaternary archives, and morphologically diagnosable hylobatid material is unavailable from most of China [16].

**Figure 1.**
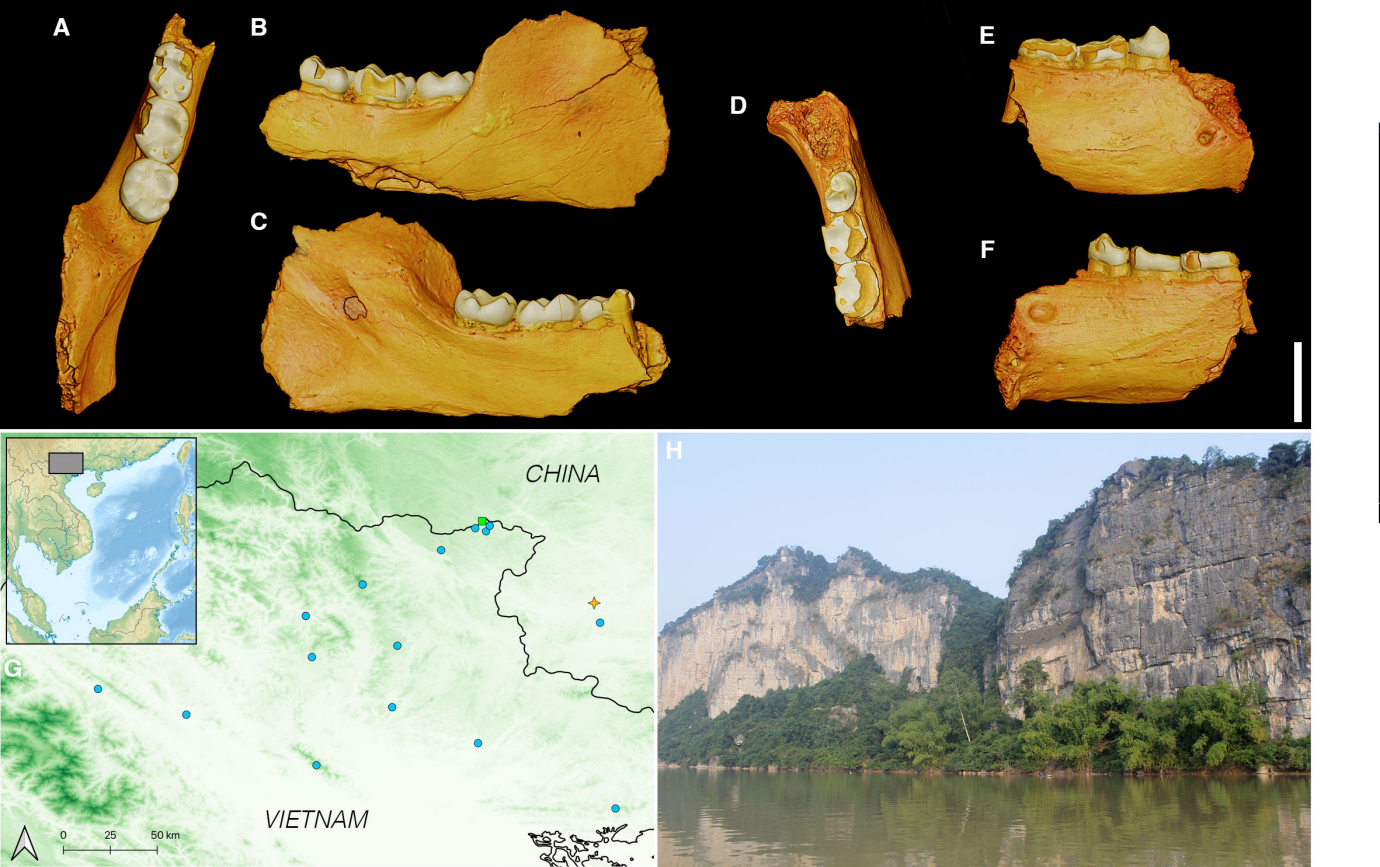
A–F, three-dimensional surface models of Wumingshan mandibles generated from micro-CT scan data. A–C, GLW-15 in occlusal, buccal and lingual views. D–F, GLW-16 in occlusal, buccal and lingual views. G, Map of southwest China and north Vietnam, showing location of Bangliang-Trung Khanh (green square), localities where *Nomascus nasutus* was extirpated in the twentieth century (blue circles), and Wumingshan (yellow star). H, Wumingshan archaeological site.

Gibbons occurred in Jingxi County (Baise Municipality) and Longzhou County (Chongzuo Municipality) in southwest China’s Guangxi Zhuang Autonomous Region until the 1950s, but were thought to be extinct before researchers could determine their species identity [26]. A remnant surviving population was discovered near the Chinese border in Vietnam’s Cao Bang Province in 2000 and was subsequently found to extend into Guangxi in Bangliang, Jingxi County [27,28] (figure 1). This represents the only extant population of the cao vit gibbon *Nomascus nasutus*, which is historically known from other sites in southwest China and north Vietnam east of the Red River [28–30]. This species is one of the world’s rarest and most threatened mammals. It is now restricted to a single *c*.50 km^2^ forest patch (the Bangliang-Trung Khanh forest block) within an inaccessible karst limestone landscape. Past censuses using traditional monitoring methods estimated a population size of *c*.120 individuals, but passive acoustic monitoring in 2021 estimated a global population of only 74 individuals [31]. Establishing a robust understanding of ecological and extinction risk parameters for this refugee species, drawing upon all available lines of evidence, is thus a priority for optimal conservation planning.

During archaeological excavations in 2013−2015, two gibbon mandibles were recovered within a Neolithic midden at Wumingshan rock shelter in Longzhou County, Guangxi, *c*.85 km from Bangliang-Trung Khanh (figure 1). Their taxonomic identity is uncertain, and they may represent either a known extant or unknown extinct gibbon. To determine the identity of these mandibles and assess what conservation-relevant information they can provide, we attempted ancient DNA extraction, conducted geometric morphometric analyses with a comparative dataset of modern and extinct hylobatids, and conducted environmental analyses to identify ecological differences associated with gibbon survival and extinction. Our combined results establish a new baseline for understanding past gibbon diversity and extinction risk, and promote the importance of using multiple complementary approaches to integrate insights from historical archives into real-world conservation problems.

## 2. Material and methods

### 2.1. Collection locality and material

Wumingshan is a 5 m wide and 20−30 m high rock shelter below a limestone cliff beside the Zuojiang River, one of a series of adjacent middle Holocene archaeological sites along this river section [32]. Accelerator mass spectrometry (AMS) dating of charcoal provides an age of 4475 ± 25 bp (Peking University ^14^C laboratory, lab number BA141169; IntCal20 calibrated date 5287−4978 bp). The Wumingshan midden contains a diverse faunal assemblage of 1626 specimens, which also includes goral, sambar and sika deer, giant and Reeves’s muntjacs, wild boar, dhole, masked and Owston’s palm civets, otter, stump-tailed macaque, brush-tailed porcupine, bamboo rat, python, turtle and fish, but lacks domesticated animals [33].

The gibbon mandibles are held in the Zooarchaeology Laboratory of Nanjing Normal University (figure 1). One specimen (GLW-15) is an incomplete left mandibular corpus with m1−3 and part of the mandibular ramus. The other specimen (GLW-16) is an incomplete right mandibular corpus with pm4, m1−2, and pm3 alveolus.

### 2.2. Genetic analysis

All pre-PCR laboratory work was conducted in the dedicated ancient DNA laboratory at the Natural History Museum, London. Bone powder (*c*.15 mg) was removed from each sample using a handheld drill (Foredom) at slow speed. DNA extraction followed the protocol of [34] with modifications: Zymo-Spin V columns were replaced with Roche High Pure Viral Nucleic Acid Large Volume spin columns, and two final elution steps of 50 μl TET buffer (total 100 μl) were performed. Dual-indexed libraries were constructed based on [35,36]. Libraries were sequenced on an Illumina NextSeq 500, producing 76-basepair paired-end reads, and were sent for mitochondrial enrichment capture at Arbor BioSciences using the myBaits^®^ MegaMito kit and sequenced on a NovaSeq S4 paired-end 150.

Sequencing reads were processed identically for both samples. Raw paired-end sequencing files were trimmed and quality-filtered using standard aDNA protocols [37]. An iterative mapping process was performed to reduce potential ascertainment bias, by conducting two independent analyses performing single-end alignment of merged trimmed reads of each sample to two different reference panels. Alignments and filtering procedures were performed using the software, commands and parameters reported in [37]. The first reference panel contained comparative full mitogenomes for all four hylobatid genera (*Hoolock*, *n* = 1; *Hylobates*, *n* = 7; *Nomascus*, *n* = 4; *Symphalangus*, *n* = 1), and the second contained six cytochrome *b* (cyt*b*) sequences, representing species from the mitogenome panel to which the samples successfully aligned reads after filtering (electronic supplementary material, table S1).

### 2.3. Morphometric analysis

To generate three-dimensional surfaces for analysis, the mandibles were scanned using a Nikon Metrology XT H 225 ST high-resolution micro-CT scanner at the Cambridge Biotomography Centre (University of Cambridge, UK) using X-Tek software (Nikon Metrology, Tring, UK). Scan parameters were: tungsten target; 0.5 mm copper filter; 120 kV; 195 mA; 1080 projections; 2 frames/projection with 1000 ms exposure. Resulting isotropic voxels were 0.017 mm^3^. Micro-CT data were reconstructed using CT-PRO 3D software (Nikon Metrology) and exported as an image (.tif) stack. Reconstructed micro-CT images were imported into Avizo (Thermo Fisher Scientific TM) and segmented using semi-automatic watershed and manual editing tools. Triangle-based surface models of tooth and bone tissues were generated using the unconstrained smoothing algorithm.

The Wumingshan specimens were compared against a large morphometric dataset of hylobatid mandibles and teeth, comprising 411 modern specimens representing 19 of the 20 extant species and all four extant genera (mean of 21.4 specimens per species, range = 1–73), three modern specimens only referrable to genus (*Hoolock* sp., *n* = 2; *Nomascus* sp., *n* = 1), and single specimens of the extinct Chinese taxa *Bunopithecus sericus* and *Junzi imperialis*. Data were collected from photographs and surface CT and micro-CT scans (electronic supplementary material, table S2). Three-dimensional digital models were oriented following the same protocols as those used for photographing original skeletal specimens, with no significant differences between data derived from digital photographs and from screenshots of three-dimensional models [23]. Sex is unknown for the Wumingshan specimens, so this variable was not included in analysis. However, gibbons do not exhibit significant sexual dimorphism in skull or dental morphology [38,39].

Taxonomic affinities of the Wumingshan specimens were investigated using two-dimensional linear measurements of molar crown size and mandibular corpus robusticity, and two-dimensional geometric morphometrics of molar crown outline and occlusal shape and mandibular corpus shape. Comparative data were collected on left mandibles and teeth when available, and metrical and shape analyses were performed exclusively on adult individuals with m3 in occlusion and no pathology. Analyses were specifically chosen to accommodate limitations arising from tooth wear or damage, and reconstruction of worn/chipped teeth and criteria for determining whether a tooth could be included in different analyses followed well-established protocols [40,41]. Analysis of crown and cervical tooth outlines with moderate to pronounced wear is less prone to misleading outcomes, whereas overall occlusal morphology is more affected by wear [42–44]: occlusal morphology analysis thus included only less worn teeth (wear stages < 2 of [45]), and more worn/damaged teeth (wear stages > 3) were only included in crown outline and metric analyses. In cases of missing or heavily damaged bone or teeth, the right antimere was used and digitally mirror-imaged (this was also conducted for GLW-16 to enable direct comparison with our dataset). Despite these protocols, each Wumingshan specimen could only be included in some analyses (electronic supplementary material, table S2).

For analysis of molar linear size data, photographs and screenshots of lower molars were collected and aligned following established protocols [24,25,46]. Standard m1−3 mesiodistal and maximum buccolingual measurements were made in Adobe Photoshop v.2.0 from scaled standardized digital images [38,47]. Except for *Hylobates klossii* m1 data [38], all data were collected by AO for consistency. Measurement data were investigated using bivariate plots of log-transformed data for individual teeth and between-group principal component analysis (bgPCA [48]) for combined m1–m2 data. Under bgPCA, the extant hylobatid sample was divided into four *a priori* genus-level groupings (*Hoolock*, *Hylobates*, *Nomascus*, S*ymphalangus*). Fossil/zooarchaeological specimens were treated as additional specimens of unknown *a priori* grouping, with their location in shape space plotted *a posteriori* on bgPC1 and bgPC2.

For analysis of molar crown outline, TpsDig [49] was used to digitize 79 equally linearly spaced points along the outline, which were slid along their curve using the criterion of minimization of bending energy [50] (electronic supplementary material, figure S1). As hylobatids exhibit considerable overlap in outline shape at species and genus level when first, second and third molars are analyzed independently [25], intra-individual outline data from m1−2 and m1−3 were combined in separate analyses to enable identification of more diagnostic shape differences across taxa (m1−2 analysis included GLW-15 and GLW-16; m1−3 analysis only included GLW-15, as m3 is not preserved in GLW-16).

For analysis of crown occlusal shape, 14 homologous landmarks were placed at tips of main cusps and intersections of main grooves and crests (electronic supplementary material, figure S1). These landmarks are useful in hominin systematics and for distinguishing various stem and crown catarrhine taxa [47,51]. Analysis only included GLW-15, as pronounced tooth wear has obliterated the occlusal morphology (e.g. cusp position, fissure pattern) of the GLW-16 molars.

Mandibular analyses followed [52]. To analyse corpus robusticity, linear height and breadth data were measured at mandibular cross-section between m1 and m2 and investigated using bivariate plots of log-transformed data. For analysis of corpus shape, cross-sectional shape between m1 and m2 was quantified by digitizing three landmarks: points on alveolar border between m1 and m2 on lingual side (landmark 1) and buccal side (landmark 2), and inferior-most point on corpus between m1 and m2 (landmark 3). Forty additional semi-landmarks were placed along the corpus outline (20 semi-landmarks between landmarks 1 and 3; 20 semi-landmarks between landmarks 2 and 3) (electronic supplementary material, figure S1). Landmarks and semi-landmarks were digitized in Avizo from scans of mandibles with little to no damage, with semi-landmarks slid along their curve using the criterion of minimization of bending energy [53]. Analyses only included GLW-16, as the mandibular body of GLW-15 is not preserved at the level of m1–m2.

For geometric morphometric analyses, coordinate data were imported into MorphoJ v.107a [54] and superimposed using generalized Procrustes analysis for conversion into shape variables. Shape variation was investigated using PCA and bgPCA, with wireframe models created to visualize extreme configurations and determine aspects of shape most correlated with PC1 and PC2. Procrustes distances were calculated at genus and species levels using principal component coordinates. Given small sample sizes for some taxa, bgPCA was not performed for combined m1−2 and m1−3 outline analyses, and m2 and m3 occlusal shape analyses were conducted at the genus level. Allometry was tested using multivariate regression of Procrustes coordinates (dependent variables) versus log centroid size (independent variable). Analyses were performed in MorphoJ, PAST v.4.07b [55] and R v.4.0.2 [56] using the packages Geomorph [57] and Morpho [58].

### 2.4. Environmental analysis

Locality records were obtained for extirpated *Nomascus nasutus* populations in southwest China and north Vietnam, with local last-occurrence dates from the 1960s–2000s [28–30,59] (*n* = 16; figure 1, electronic supplementary material, table S3). Other regional records that may represent *N. nasutus*, including nearly all Chinese records, were excluded due to (i) non-specific localities (e.g. reported only at county, municipality or province level) in twentieth-century or older reports [15,17,26]; (ii) taxonomic uncertainty [29]; (iii) morphometric analysis indicating that Pleistocene fossils from Guangxi probably represent a distinct, now-extinct species [25].

Data were collected on environmental conditions (bioclimatic, abiotic, anthropogenic) for Bangliang-Trung Khanh, historical *N. nasutus* localities and Wumingshan. Climate data for 1970−2000 were obtained from Bangliang-Trung Khanh and historical localities at *c*.1 km (0.5 arc-minute) resolution from WorldClim v.2.1 [60], and mid-Holocene (Northgrippian) climate data for Wumingshan were obtained at *c*.5 km resolution from PaleoClim v.1.0.1 [61]. Elevation data were obtained at 90 m resolution from the SRTM Digital Elevation Model [62], and slope data were calculated from this dataset using the ‘terra’ package in R v.4.4.0. Geology data were obtained from the Global Lithological Model [63], with 16 geology types converted from vectors into separate percentage-cover rasters. Global Human Footprint (GHF) data for 1995−2004 were obtained at 1 km resolution [64]. All layers were reprojected into ESRI:102028-WGS 1984 Albers Equal Area for Southern Asia to standardize pixel areas, resampled to 50 km^2^ to match the approximate area occupied by the surviving *N. nasutus* population [30], and aligned. Geology layers containing fewer than three datapoints were removed. Variables were tested for multicollinearity using variance inflation factors (VIF), with removal of one of each pair of variables with a VIF score above 7 (the variable from each pair that was also correlated with more additional variables was removed). The final dataset contained five bioclimatic variables (BIO2: Mean Diurnal Temperature Range; BIO4: Temperature Seasonality; BIO12: Annual Precipitation; BIO15: Precipitation Seasonality; BIO18: Precipitation of Warmest Quarter), elevation, slope, geology and GHF. For historical records from protected areas above 50 km^2^ in areas with no further locality details, environmental values were averaged across all pixels within the area.

A minimum convex polygon was made around all records, with a 50 km buffer. Comparative data on environmental conditions were sampled for all pixels lacking gibbon records within this polygon (*n* = 1848). Abiotic and GHF values for all sites where *N. nasutus* has been extirpated (twentieth-century sites and Wumingshan) were compared with values for pixels lacking gibbon records using two-tailed two-sample *t*-tests, and values for Bangliang-Trung Khanh were compared with values for background points using two-tailed one-sample *t*-tests. All environmental values for Bangliang-Trung Khanh and Wumingshan were also compared with values for other combined *N. nasutus* sites using two-tailed one-sample *t*-tests. Statistical significance was set at *p* = 0.0025 following Bonferroni correction. Following standardization of variables, a PCA was also conducted to investigate how modern, historical and zooarchaeological *N. nasutus* sites are distributed in environmental niche space in relation to background points.

## 3. Results

### 3.1. Genetic analysis

Unfortunately, neither specimen yielded sufficient DNA to enable downstream analysis. After duplicate removal, GLW-15 had only 0−17 aligned reads to the reference mitochondrial genomes and 1−2 aligned reads to the cyt*b* reference panel, and GLW-16 had only 1−8 and 0 aligned reads, respectively.

### 3.2. Morphometric analysis

The Wumingshan specimens are characterized by lower molars with subrectangular crowns (mesiodistally longer than broad), with peripherally placed cusps and limited to moderate buccolingual waisting along the mesiodistal axis. The buccal cusps are slightly mesial to the lingual cusps, and the hypoconulid is buccocentrally located relative to the crown longitudinal midline. The protoconid and hypoconid are low and rounded, and the metaconid and entoconid are more elevated and pyramidal-shaped. The metaconid is the largest cusp in all molars, followed by the protoconid and hypoconid, which are approximately equal in size. The lingual cristids are sharper than the buccal cristids, and the hypoprotocristid and hypometacristid are well-defined. The crown outline shows well-rounded corners, with symmetrical mesial arching of the preprotocristid and premetacristid. The mesial fovea is rectangular and intermediate in size, whereas the distal fovea is smaller and ill-defined. There is no trace of a buccal cingulid. A simple Y-groove pattern is present. The basin is smooth and expansive with no wrinkling. The buccal and lingual walls are bulging, and the m3 shows minimal distal tapering. The mandible exhibits a tall, narrow corpus that tapers inferiorly and extends laterally. Tooth measurements in comparison to other hylobatids are given in electronic supplementary material, table S4.

**Figure 2.**
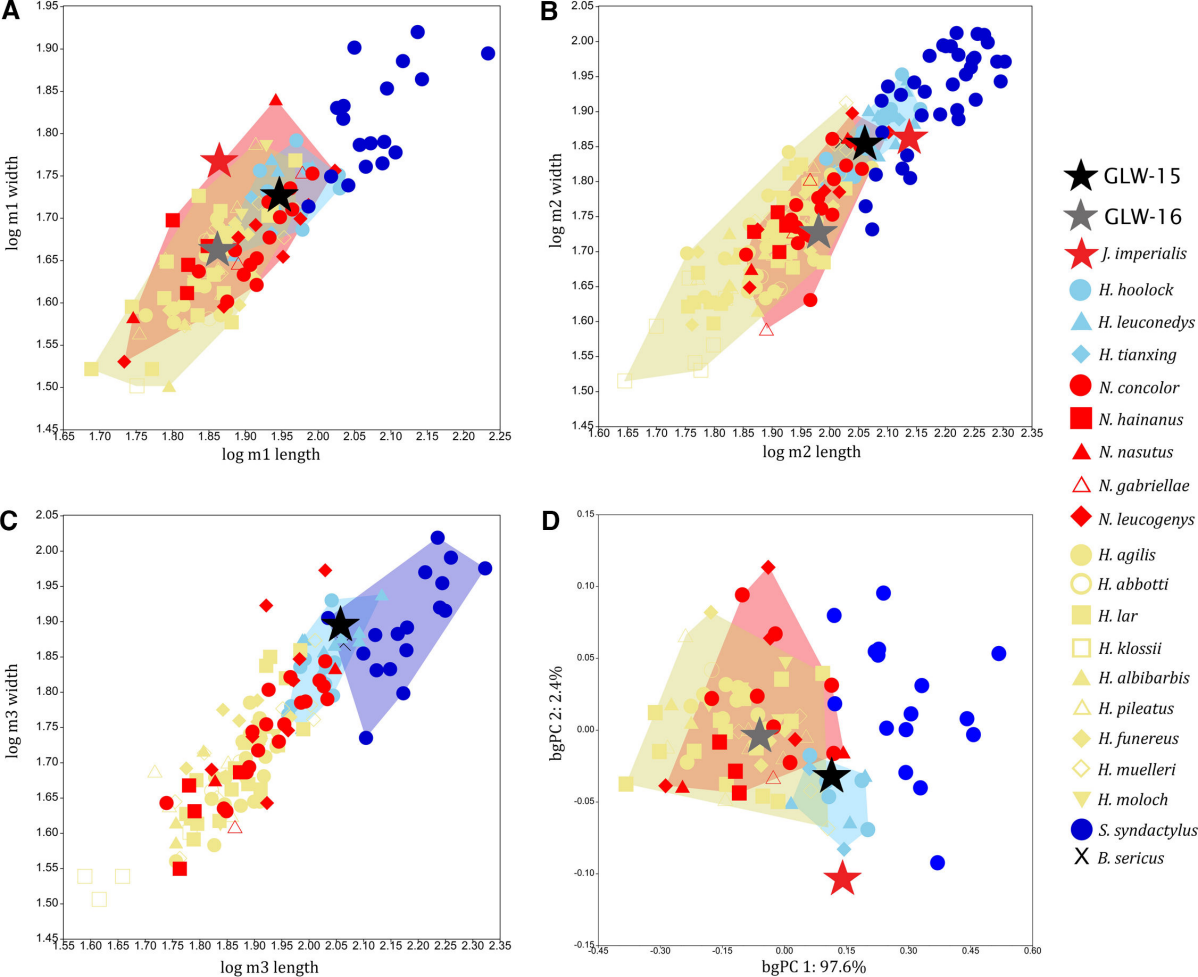
Bivariate plots of log-transformed lower molar crown mesiodistal length against buccolingual width: A, m1 (including GLW-15 and GLW-16); B, m2 (including GLW-15 and GLW-16); C, m3 (including GLW-15 only). D, bgPCA of combined m1-m2 measurement data (only specimens with both m1 and m2 included, including GLW-15 and GLW-16). Plots show convex hulls of extant hylobatid taxa that overlap with Wumingshan specimens.

When molar length is plotted against width, GLW-15 m1 and m2 fall within the variation range of *Hoolock*, *Hylobates* and *Nomascus*, GLW-16 m1 and m2 fall within *Hylobates* and *Nomascus*, and GLW-15 m3 falls within *Hoolock* and *Symphalangus*. For bgPCA of combined m1–m2 measurement data, GLW-15 falls within *Hoolock* and is also close to both *Hylobates* and *Nomascus*, and GLW-16 falls within *Hylobates* and *Nomascus* (figure 2) (electronic supplementary material, table S5).

**Figure 3.**
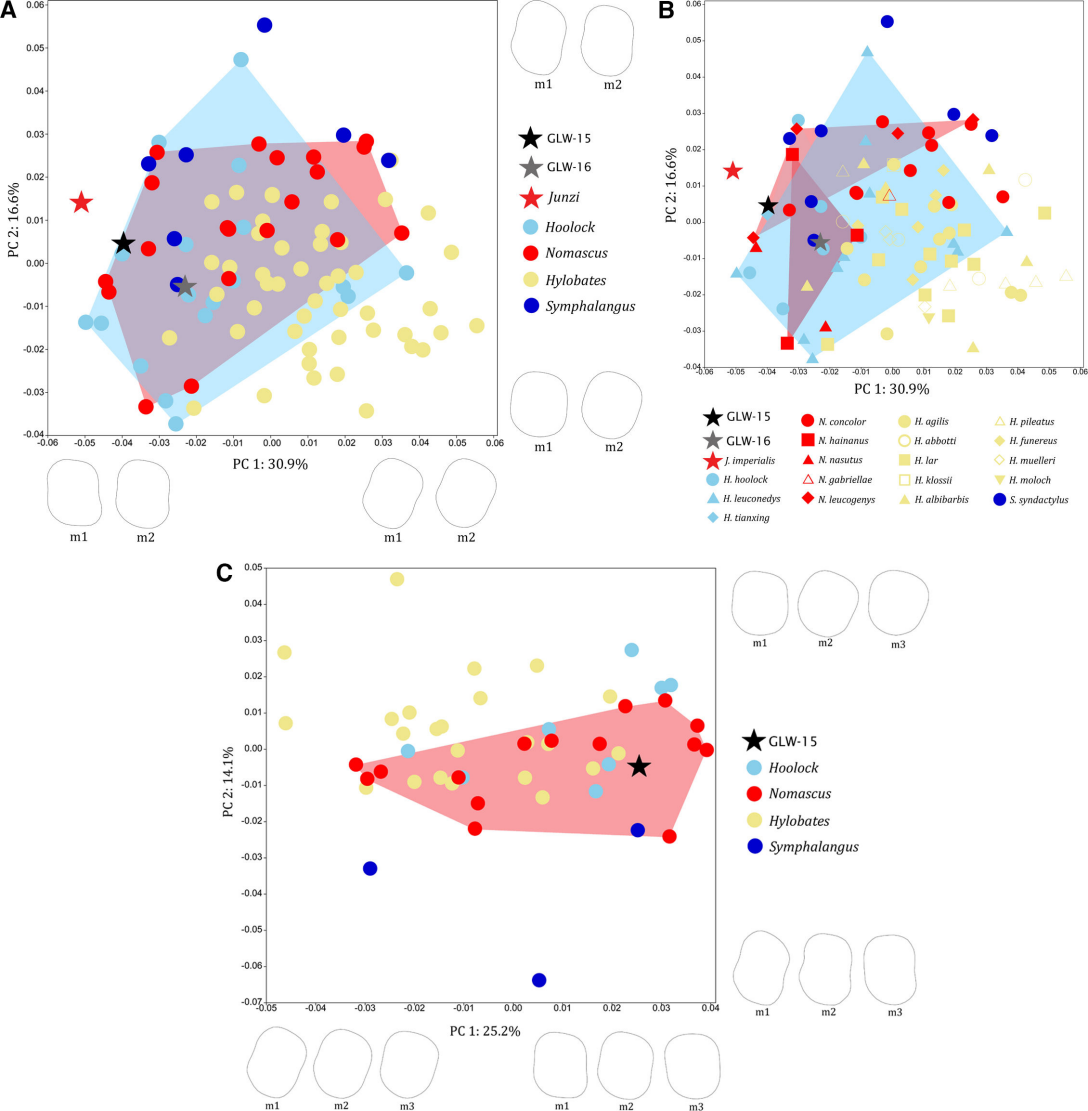
PCAs of combined lower molar crown outline data. A, genus-level variation in m1–m2 (including GLW-15 and GLW-16); B, species-level variation in m1–m2 (including GLW-15 and GLW-16); C, genus-level variation in m1–m3 (including GLW-15 only). Plots show convex hulls of extant hylobatid taxa that overlap with Wumingshan specimens. Wireframes illustrate shape changes of m1–m2 and m1–m3 crown outline along PC1 and PC2 in occlusal view (left molars depicted, lingual aspect to the right).

In analyses of combined m1–m2 outline data, GLW-15 falls within genus-level variation of *Hoolock* and *Nomascus*, and species-level variation of *N. leucogenys*; and GLW-16 falls within genus-level variation of *Hylobates* and *Nomascus*, and species-level variation of *Ho. leuconedys* and *N. hainanus* (figure 3A,B). For combined m1–m3 outline data, GLW-15 falls exclusively within genus-level variation of *Nomascus*, and species-level variation of *N. concolor* and *N. hainanus* (figure 3C). When all principal components are considered, average combined pairwise Procrustes distances for GLW-15 m1−2 are closest to *Ho. hoolock*, *N. hainanus* and *N. nasutus*, GLW-15 m1−3 are closest to *N. hainanus*, *Ho. hoolock*, *N. nasutus*, *N. concolor* and *N. leucogenys*, and GLW-16 m1−2 are closest to *Ho. hoolock*, *Ho. leuconedys*, *N. nasustus* and *N. hainanus*. Most permutation tests for between-group Procrustes distances differentiate between extant hylobatids, but distances of GLW-15 and GLW-16 with extant groups are non-significant (electronic supplementary material, table S5). Relationships between Procrustes coordinates and log centroid size are all significant (*p* < 0.05), with size explaining 1.94, 2.46 and 3.84% of shape variance for m1, m2 and m3, respectively.

**Figure 4.**
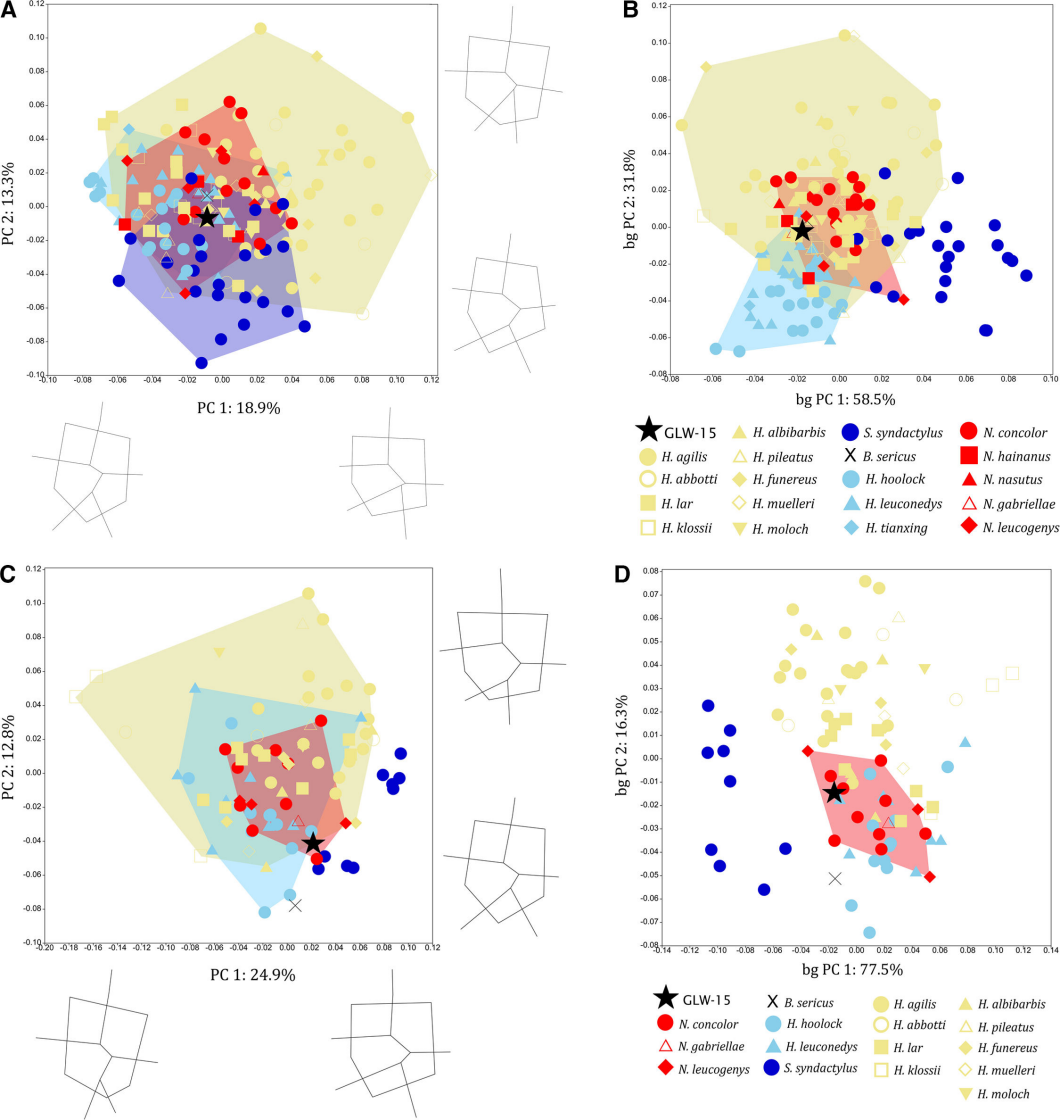
Analyses of species-level variation in lower molar occlusal shape, including GLW-15 only: A, m2 PCA; B, m2 bgPCA; C, m3 PCA; D, m3 bgPCA. Plots show convex hulls of extant hylobatid taxa that overlap with GLW-15. Wireframes illustrate shape changes of m2 (A) and m3 (C) occlusal shape along PC1 and PC2 in occlusal view (left molars depicted, lingual aspect to the right).

In molar occlusal shape analyses including GLW-15, m2 falls within the variation range of all four extant hylobatid genera in standard PCA and within *Hylobates*, *Hoolock* and *Nomascus* in bgPCA (figure 4A,B), and m3 falls within *Hylobates*, *Hoolock* and *Nomascus* in PCA and exclusively within *Nomascus* in bgPCA (figure 4C,D). Although most permutation tests for between-group Procrustes distances differentiate between extant hylobatids, distances for GLW-15 m2 and m3 occlusal shape are non-significant, but show closest affinities to *Nomascus* (electronic supplementary material, table S5). Relationships between Procrustes coordinates and log centroid size are all significant (*p* < 0.05), with size explaining 5.25% of shape variance for m2 and 8.35% for m3.

**Figure 5.**
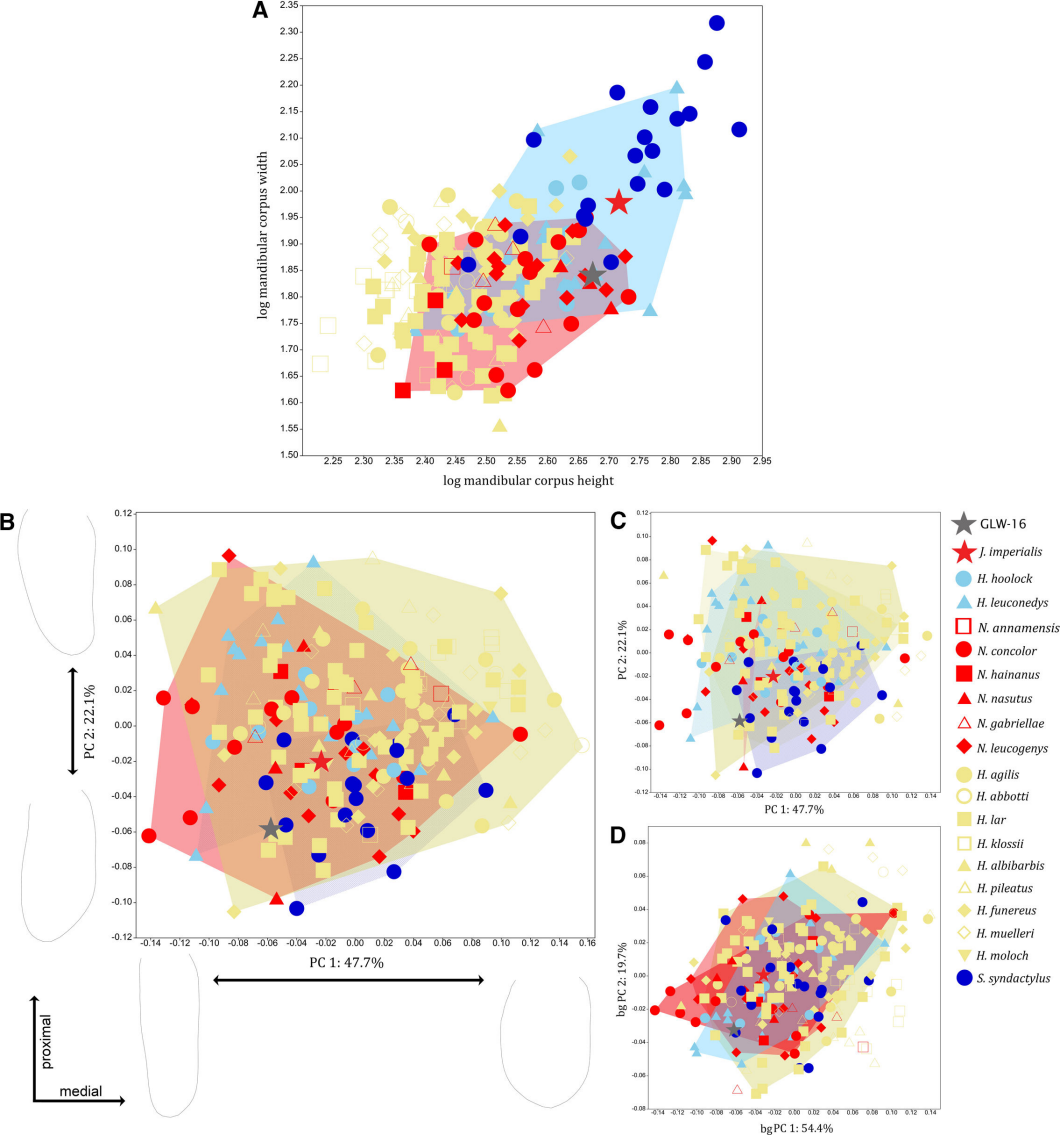
Analyses of mandibular shape, including GLW-16 only. A, bivariate plot of log-transformed mandibular corpus height against corpus width. B–D, analyses of variation in mandibular corpus shape: B, genus-level PCA; C, species-level PCA; D, species-level bgPCA. Plots show convex hulls of extant hylobatid taxa that overlap with GLW-16.

In mandibular analyses, GLW-16 falls within the variation range of *Hoolock* and *Nomascus* for robusticity (figure 5A). Corpus shape variation shows considerable between-group overlap along the first two principal components, with GLW-16 falling within *Hylobates* and *Nomascus* at the genus-level within PCA; within *Hy. funereus* and *Hy. lar* and close to *Ho. leuconedys*, *N. nasutus* and *S. syndactylus* at the species-level within PCA; and within *Ho. leuconedys*, *Hy. lar*, *N. concolor* and *N. leucogenys* at the species level within bgPCA (figure 5B–D). GLW-16 exhibits negative scores along PC1 (47.7% of variance, associated with taller corpus that is relatively narrower throughout cross-section) and PC2 (22.1% of variance, associated with corpus of intermediate width tapering inferiorly towards buccal/lateral). Most permutation tests for between-group Procrustes distances in corpus shape differentiate between extant hylobatids; GLW-16 shows the closest distance to *Nomascus*, and specifically *N. nasutus* (electronic supplementary material, table S5). The relationship between Procrustes coordinates and log centroid size is non-significant across the sample, with size explaining only 1.01% of shape variance (*p* = 0.0744), but is significant for within-genus variation, with size explaining 1.25% of variance (*p* < 0.05).

### 3.3. Environmental analysis

Sites where *N. nasutus* has been extirpated do not differ from background points in elevation (*p* = 0.283), slope (*p* = 0.069) or GHF (*p* = 0.781). Conversely, Bangliang-Trung Khanh differs significantly from background points in elevation (Bangliang-Trung Khanh mean: 786.7 m, background mean: 417.8 m, *p* < 0.0001), slope (Bangliang-Trung Khanh mean: 25.2 degrees, background mean: 14.3 degrees, *p* < 0.0001) and GHF (Bangliang-Trung Khanh mean: 25.3, background mean: 33.2, *p* < 0.0001). Only 1.8% of background points show combined values of the same or higher elevations and slopes and same or lower GHF.

Compared to sites where *N. nasutus* survived until recent decades (Bangliang-Trung Khanh and twentieth-century sites), Wumingshan differs in all bioclimatic variables except BIO2 (BIO4, Wumingshan mean: 6121.3, other sites mean: 4799.3, *p* < 0.0001; BIO12, Wumingshan mean: 1269.4, other sites mean: 1644.0, *p* < 0.0001; BIO15, Wumingshan mean: 76.2, other sites mean: 85.0, *p* < 0.0001; BIO18, Wumingshan mean: 616.6, other sites mean: 928.1, *p* < 0.0001). Wumingshan also differs from sites where *N. nasutus* survived later in elevation (Wumingshan mean: 114.7 m, other sites mean: 534.6 m, *p* < 0.0001) and slope (Wumingshan mean: 1.99, other sites mean: 19.2, *p* < 0.0001), but with no difference in GHF (*p* = 0.183). Compared to sites where *N. nasutus* is extirpated (twentieth-century sites and Wumingshan), Bangliang-Trung Khanh does not differ in bioclimatic variables, but differs in elevation (modern site mean: 786.7 m, other sites mean: 497.3 m, *p* = 0.0008), slope (modern site mean: 25.2, other sites mean: 17.9, *p* = 0.001), and GHF (modern site mean: 25.3, other sites mean: 32.7, *p* = 0.0002). Bangliang-Trung Khanh and Wumingshan are both entirely composed of carbonate sedimentary rock, whereas other *N. nasutus* sites generally contain less carbonate sedimentary rock (mean: 64.8%, range: 0–100%) and more mixed sedimentary rock (mean: 22.8%, range: 0–96%).

**Figure 6.**
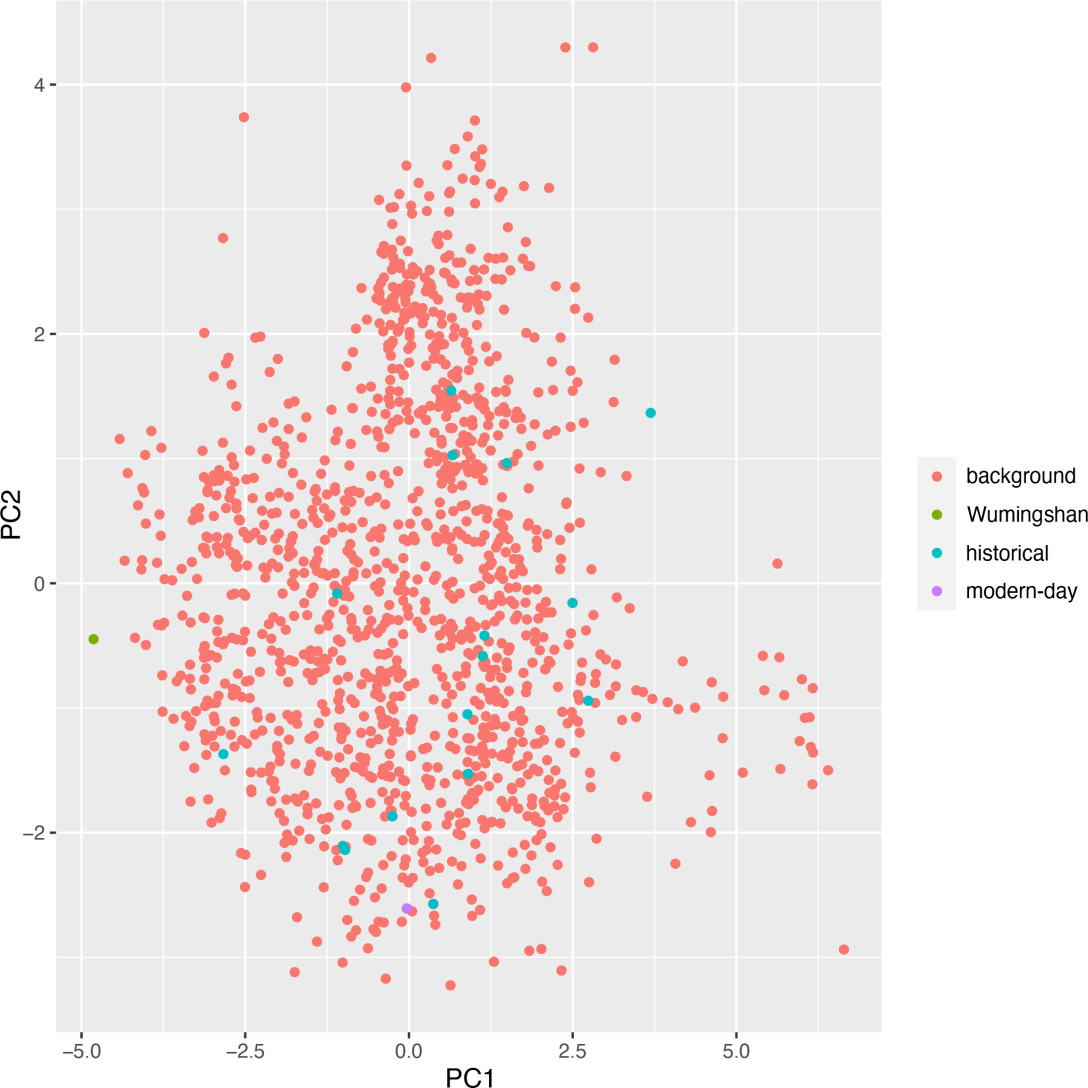
Principal component analysis (PC1-PC2) of modern, historical and zooarchaeological *N. nasutus* localities in environmental niche space (including bioclimatic, abiotic and anthropogenic variables) in relation to background points.

In PCA, the first three principal components have eigenvalue scores above 1 and together account for 71.4% of total environmental variation across locality points (PC1, 33.6%; PC2, 22.0%; PC3, 15.8%). Wumingshan is separated from all modern and historical gibbon localities along PC1, where the greatest loadings (>0.4) are associated with the bioclimatic variables BIO18 (0.501), BIO12 (0.470), BIO15 (0.451) and BIO4 (−0.424). Wumingshan is associated with lower precipitation amount and seasonality, lower diurnal temperature range, and higher temperature seasonality along PC1. Bangliang-Trung Khanh is situated just outside all other gibbon localities along PC2, where the greatest loadings are instead associated with landscape structure (slope: −0.459; presence of carbonate sedimentary rock: −0.452; elevation: −0.451) and GHF (0.446). Bangliang-Trung Khanh is associated with higher slope, more carbonate sedimentary rock, higher elevation, and lower GHF along PC2 (figure 6). Neither Wumingshan nor Bangliang-Trung Khanh are well-differentiated from other gibbon localities along PC3, where the greatest loadings are associated with presence of mixed sedimentary rock (−0.671) or carbonate sedimentary rock (0.501) (electronic supplementary material, figure S2).

## 4. Discussion

### 4.1. Taxonomic assignment of Wumingshan mandibles

Molecular evidence was unfortunately unavailable to make taxonomic inferences, and our morphometric analyses of molar and mandibular characteristics provided differing signals and resolutions for the taxonomic affinities of the Wumingshan mandibles. In most analyses, both specimens fell within the variation range for two and sometimes three extant hylobatid genera, and GLW-15 fell within the variation of all extant genera in m2 occlusal shape analysis.

It is possible that this reduced discriminatory ability reflects limited phylogenetic differentiation in dental and mandibular morphology across hylobatids (at least for the regions preserved in our specimens), or conflicting morphological patterns associated with evolutionary convergence as well as evolutionary history. Hylobatids represent a recent evolutionary radiation, with all extant genera having diverged *c*.5 million years ago [65], and with species-level divergences from the Late Pliocene to Late Pleistocene [66]. Hylobatid taxonomy has undergone substantial recent revision at both species and genus levels [67–69], with species largely differentiated on soft-tissue characteristics (e.g. pelage coloration) and behavioural characteristics (e.g. vocalizations) rather than external or skeletal measurement data [67,70]. Craniodental characteristics are also influenced by both phylogeny and functional adaptation [71,72], and although geometric morphometric analysis of primate molar shape demonstrates a strong phylogenetic signal at higher taxonomic levels [73], between-species morphological differences across primates are often obscured by homoplasy associated with variation in adaptive dietary ecology [74–76]. Although hylobatids are mainly ripe-fruit specialists that use figs as fallback resources, different taxa vary in their fruit-pulp specialism, fig-eating and folivory, such as across habitats with varying seasonality [77], indicating the potential for functional variation in dental and mandibular characteristics [78,79].

Whereas molar metrics can successfully differentiate between genera, species and subspecies of great apes [80], comparable studies have been unable to differentiate reliably between hylobatid taxa, especially at lower taxonomic levels [81,82]. Landmark-based shape analysis has shown that mandibular corpus outline carries a strong taxonomic signal among large-bodied apes [52], which can be of great value when dealing with fragmentary material such as the Wumingshan mandibles. However, the usefulness of the ratio of corpus breadth and height for capturing mandibular morphology in hominoids and other primates has been questioned due to positive allometry [83,84], and this approach also only captures variation in cross-sectional shape of a complex three-dimensional anatomical structure. Indeed, differentiation among hylobatid genera on the basis of mandibular corpus morphology alone appears to be particularly challenging, as all hylobatid species show gracile cross-sections [84]. Despite these limitations, craniodental morphometric analyses using large multi-character datasets have demonstrated that gibbon genera and species can be differentiated statistically, despite overlap in trait variation across morphospace [23–25,69]. Indeed, our approach to combine data for multiple molars from the same individuals in crown outline analyses is shown to improve sensitivity for differentiating hylobatid taxa [25]. Previous geometric morphometric investigations also show that two-dimensional datasets can have better species-level discrimination than three-dimensional datasets for primate molar analysis. This may be because cusp height is associated with homoplastic between-species differences in foraging ecology and thus risks obscuring high-resolution phylogenetic signal [85], or alternately because adding a third dimension decreases the signal-to-noise ratio.

We therefore consider that the reduced discriminatory ability of our analyses is instead probably caused by sampling error associated with small sample sizes, reflecting the fact that many extant hylobatids are poorly represented in museum collections due to their rarity and/or historical inaccessibility of their habitats, and providing a limited understanding of dental and mandibular variation. Data limitation is particularly problematic for species that occur in southwest China (*Ho. tianxing*, *N. hainanus*, *N. nasutus*), and for understanding morphometric variation across *Nomascus*, for which one of the seven species is absent from our dataset (*N. siki*), and four are represented by only 1−6 individuals (*N. annamensis*, *N. gabriellae*, *N. hainanus*, *N. nasutus*). These limited samples are also not necessarily uniform in size or morphology. Although mammal species are often identifiable using cranial geometric morphometrics even with relatively small sample sizes, taxonomic assignment errors are frequent with fewer specimens, and primates in particular are prone to inaccuracies [86]. Sample sizes of over 20 individuals per species have thus been recommended to capture intraspecific variation [86]. Taxonomic uncertainty is also shown in morphometric attempts to identify zooarchaeological primate material using limited modern comparative samples [85].

Despite the unavoidable shortcomings of this data limitation, our study represents the best attempt to identify the Wumingshan mandibles, by conducting multiple analyses using dental and mandibular characters and both linear and geometric morphometric frameworks. Our results demonstrate that, while morphometric relationships to other genera differ across analyses, the specimens almost always fall within or very close to variation shown by our available *Nomascus* sample in genus-level analyses, and sometimes within *Nomascus* to the exclusion of other genera (m1–m3 molar outline analysis; m3 occlusal shape bgPCA). Furthermore, both specimens fall within or very close to Chinese *Nomascus* species (*N. concolor*, *N. hainanus*, *N. leucogenys*, *N. nasutus*) in all species-level analyses. Despite the fact that the spread of *Nomascus* in morphospace is probably artificially limited by the very small samples of most species, both Wumingshan specimens exhibit a close relationship to this genus across almost all analyses, and the balance of evidence indicates that they can both be assigned to *Nomascus*. Specifically, they share the following characteristics with *Nomascus*: (i) subrectangular molars that are fairly symmetrical (rather than skewed) along the longitudinal axis; (ii) limited waisting and no distal tapering; (iii) intermediate-sized molars with moderate to pronounced size differences between m1 and m2, and with m3 larger than m2; (iv) protoconid and hypoconid set slightly mesial relative to metaconid and entoconid, respectively (GLW-15); (v) a buccocentrally located hypoconulid relative to longitudinal midline of crown (GLW-15); (vi) a tall and narrow mandibular corpus that tapers inferiorly and laterally (GLW-16). While each characteristic might also be present in other hylobatids, especially *Hoolock* species, this combination occurs almost exclusively in *Nomascus*. The GLW-16 mandibular corpus in particular is strikingly similar to the condition seen in modern *N. nasutus* specimens.

Although some Asian landscapes support multiple co-occurring hylobatid genera, species within each genus have non-overlapping distributions, with allopatric ranges delimited by river systems or other barriers, and with only one *Nomascus* species present in any landscape [67,87]. Wumingshan is within the known historical distribution of *N. nasutus* (figure 1), and close to Longgang National Nature Reserve, where the species persisted into the twentieth century [29]. While it is difficult to be fully confident about the species-level diagnosis of the Wumingshan mandibles based upon morphology alone, we therefore refer both specimens to *N. nasutus* based upon the evidence provided by both morphology and biogeography.

### 4.2. Spatiotemporal extinction dynamics and ecological refugia

Today, *N. nasutus* survives as a tiny population in one geographically restricted location, meaning that conservation planning based solely upon modern-day data cannot determine the species’ wider range of ecological tolerances, or contextualize whether environmental conditions within this refugium are ecologically optimal or marginal. We were unable to investigate vegetational characteristics directly between Bangliang-Trung Khanh and sites where *N. nasutus* has been lost, due to potential change in forest structure and landscape conditions at other sites through human activity and Holocene climatic shifts [61,88]. However, our comparative assessment of bioclimatic, abiotic and anthropogenic parameters establishes a new baseline for understanding the refugee status, environmental niche, extinction vulnerability, and management requirements of *N. nasutus*.

Sites where *N. nasutus* formerly occurred do not differ from background points across the same region in landscape structure (elevation or slope). Furthermore, Wumingshan, where the species was extirpated before the recent historical period, has lower elevation, lower slope, and a different range of bioclimatic parameters (greater temperature seasonality, reduced diurnal temperature range, reduced precipitation indices and seasonality) compared to sites where the species persisted within living memory. These differences are shown by statistical comparisons and/or by the greatest loadings for PC1 in our PCA, where Wumingshan is clearly differentiated from all other sites. These findings demonstrate that *N. nasutus* formerly occurred over a much wider range of bioclimatic and abiotic conditions, and thus potentially constitutes a landscape-generalist species. In contrast, our statistical and PCA results show Bangliang-Trung Khanh represents a high-elevation refugium (higher elevation and slope) within the species’ former range, and is also significantly more inaccessible in terms of both elevation and slope compared to all sites where local *N. nasutus* populations have been extirpated. Conversely, Bangliang-Trung Khanh is no different in bioclimatic parameters compared to sites where the species has been lost, indicating the importance of local landscape structure for providing an ecological refuge. This finding is supported by spatial relationships between gibbon records and GHF, with *N. nasutus* lost from all sites where local human pressures are typical for the wider region, and only surviving at a site with significantly low GHF, which is likely correlated with local landscape inaccessibility.

These findings demonstrate that the dynamic biogeography of past population decline in *N. nasutus* is comparable to that of many other threatened species [5–9], including Chinese gibbons [17,89], which were former landscape generalists but now persist only as high-elevation refugee populations. Previous studies of historical gibbon and other mammalian extinction dynamics demonstrate that extinctions across eastern and southeast Asia show strong spatial structuring, indicating that population persistence or loss was regulated by anthropogenic pressures that spread directionally across the region [90]. Multiple species, including other regionally extirpated mammals present in the Wumingshan deposit [18,19], experienced progressive range contraction towards the southwest in response to a ‘wavefront’ of human demographic expansion associated with increased forest loss and hunting [88]. However, at the finer geographic scale of our study region, *N. nasutus* populations show no obvious spatial pattern of survival or loss across sites, suggesting that local-scale persistence is further regulated by specific landscape and environmental parameters. Similar patterns of complex regional decline, fragmentation and isolation are also seen in several other species, including gibbons, in other systems, and reflect the distribution of specific refugial conditions that limit local human access [89,91,92].

Our results provide important further insights into the conditions required for persistence of threatened populations. In comparison to other historical *N. nasutus* sites, which vary in their local geology, the Bangliang-Trung Khanh and Wumingshan landscapes both consist entirely of limestone (figure 1). This landscape structure is thought to have contributed to site inaccessibility and gibbon survival at Bangliang-Trung Khanh [30], which is differentiated from other gibbon localities along PC2 partly on the basis of presence of carbonate sedimentary rock. Similar karst systems are known to act as important refugia for populations of many other species across southeast Asia [93]. However, the geologically similar landscape at Wumingshan lost its gibbons before recent history, indicating that the structural features of limestone landforms alone are insufficient to ensure gibbon survival within human-occupied systems. Instead, a combination of landscape characteristics (higher elevation and slope as well as suitable geological landforms) may be required to occur together to support local gibbon persistence in the absence of conservation management.

Together, our analyses provide a new baseline for considering conservation actions for *N. nasutus*. Bangliang-Trung Khanh does not differ bioclimatically from other sites that historically supported gibbons, so it does not obviously represent suboptimal niche space in regard to these environmental parameters. However, the site is at the upper margin of the species’ elevational range, and elevation influences key ecological characteristics of tropical forests (e.g. net primary productivity) in potentially complex ways [94]. We recommend further investigation into potentially atypical behavioural, ecological or demographic characteristics (e.g. group size, home range size) exhibited by *N. nasutus* at Bangliang-Trung Khanh associated with persistence under high-elevation conditions [95]. In terms of wider-scale conservation planning, modern-day data alone would suggest there are few suitable sites left across the species’ former range that share key environmental characteristics with Bangliang-Trung Khanh (only 1.8% of background points have a comparable set of elevation, slope and GHF threshold values), meaning that few other places might thus be able to support another population. However, older records reveal a much greater range of environmental tolerances and potential conservation landscapes across the species’ former extent of occurrence, raising the possibility of population restoration through translocation to other suitable sites. This is an important concern, as the Bangliang-Trung Khanh population may be approaching the site’s carrying capacity, necessitating evidence-based identification of novel conservation solutions to aid species recovery by increasing population size and distribution [96]. Past ecological baselines also indicate that *N. nasutus* may have the capacity for greater resilience to climatic change than suggested by modern-only data, although this requires further investigation through modelling habitat and resource shifts (e.g. in key food plants [97]) and associated changes in landscape use by local communities.

### 4.3. Conclusions

Our multidisciplinary approach, comprising morphometric and ecological investigations of past and present specimens and data, establishes a new perspective on niche requirements and population change for one of the world’s rarest mammals and highlights potential new directions for its conservation. We recommend the adoption of similar combined methods to generate novel conservation evidence for other threatened species using subfossil and zooarchaeological material. Through this approach, we encourage wider integration of environmental archives into modern biodiversity research and management under the emerging discipline of conservation palaeobiology [98], in recognition of the urgent need to expand the conservation toolkit in order to mitigate the global extinction crisis.

## Data Availability

Morphometric landmark data used in this study are available online at University College London’s Research Data Repository. All additional data are provided in the electronic supplementary material.
